# Supplementary material for: Microglial metabolism is a pivotal factor in sexual dimorphism in Alzheimer’s disease
Source: Commun Biol. 2021 Jun 10;4:711. doi: 10.1038/s42003-021-02259-y (PMC8192523; doi:10.1038/s42003-021-02259-y)
Supplement: Supplementary file 2 — Supplementary Information [file 42003_2021_2259_MOESM2_ESM.pdf]

Supplementary Table 1

Non-demented controls						
Donor	Sex	Age	Brain weight	Pmd	Braak Score	Brain area
1995-006	M	81	1274	505	1	H & PC
**1996-052**	**M**	**73**	**1500**	**550**	**2**	**H**
1998-126	M	71	1345	360	2	H & PC
2017-016	M	72	1385	260	2	H & PC
1995-110	F	81	1022	1335	1	H & PC
2011-028	F	81	1075	265	1	H & PC
2015-034	F	82	1318	465	1	H & PC
2015-087	F	75	1305	550	1	H & PC
**1997-163**	**M**	**74**	**1328**	**353**	**2**	**PC**
Alzheimer's disease						
1994-086	M	75	1098	330	5	H & PC
2000-042	M	78	1208	465	5	H & PC
2001-092	M	79	1118	305	5	H & PC
2007-025	M	82	1182	315	5	H & PC
2007-078	M	77	1104	270	5	H & PC
1992-025	F	80	1046	265	5	H & PC
1992-069	F	78	889	265	5	H & PC
1992-100	F	77	1016	252	5	H & PC
1994-083	F	75	960	255	5	H & PC

Details of hippocampal and parietal samples obtained from the Netherlands brain bank. Samples were obtained from the same individuals except Donor 1996-052 (hippocampus) was replaced by Donor 1997-163 (parietal cortex); these samples are highlighted in bold text

Abbreviations: Pmd=post-mortem delay, H=hippocampus; PC= parietal cortex

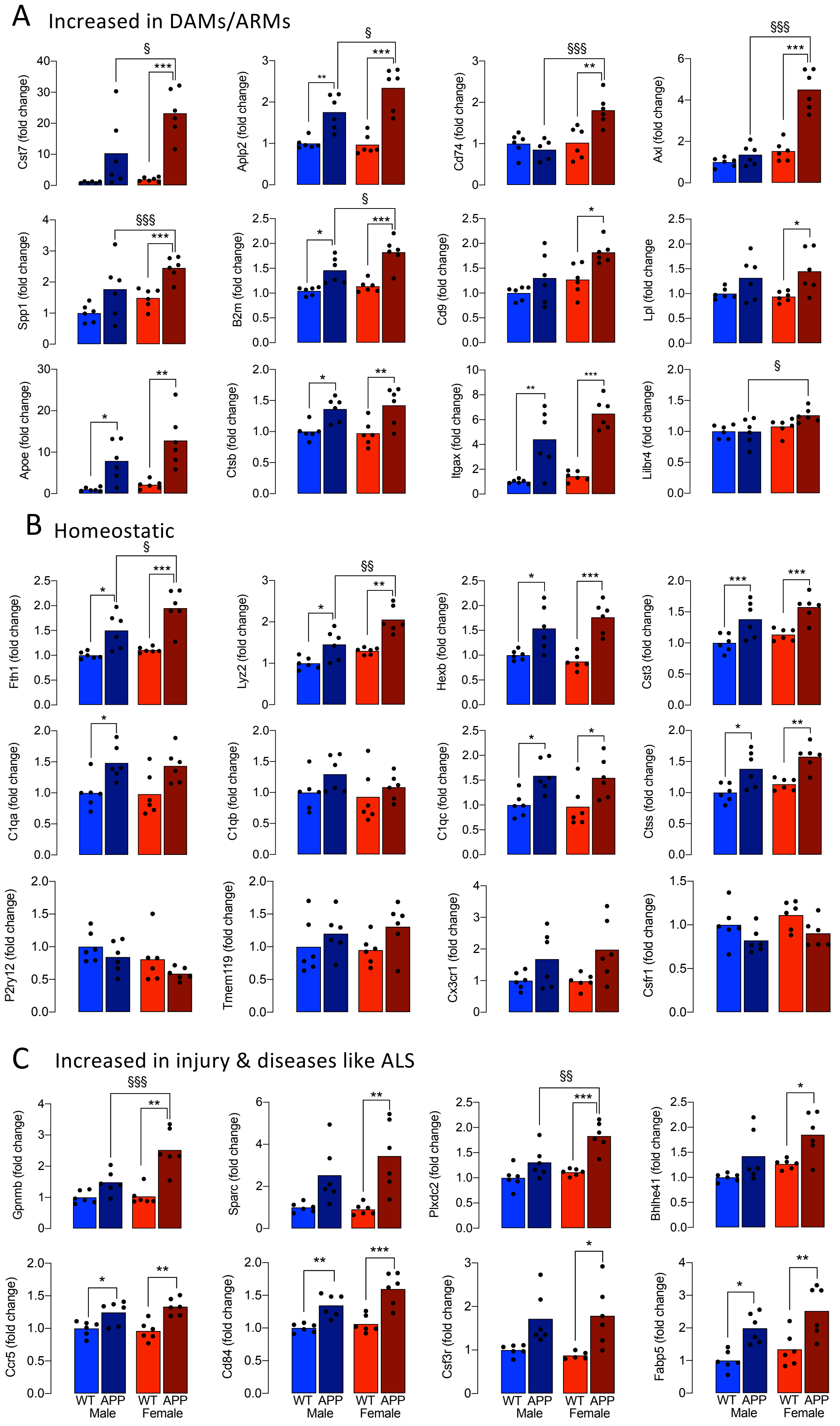

Supplementary Figure 1. Expression of genes associated with microglial activation status in APP/PS1 mice is sex-related. Significant genotype x sex interactions were observed in *Axl* ($p < 0.001$), *Cd74, Spp1* ($p < 0.01$), *Cst7, Aplp2* and *Plxdc2* ($p < 0.05$). Post hoc analysis revealed significant increases in *Cst7, Aplp2, Cd74, Axl, Spp1, B2m, Lilbr4, Fth1, Lyz2, Gpnmb* and *Plxdc2* in APP/PS1 female, compared with APP/PS1 male, mice ($^{\S}p < 0.05$; $^{\S\S}p < 0.01$; $^{\S\S\S}p < 0.001$). Significant genotype-related increases were observed in several genes in APP/PS1 male, compared with WT male, mice and in APP/PS1 female, compared with WT female, mice as indicated by the asterisks (*$p < 0.05$; **$p < 0.01$; ***$p < 0.001$).

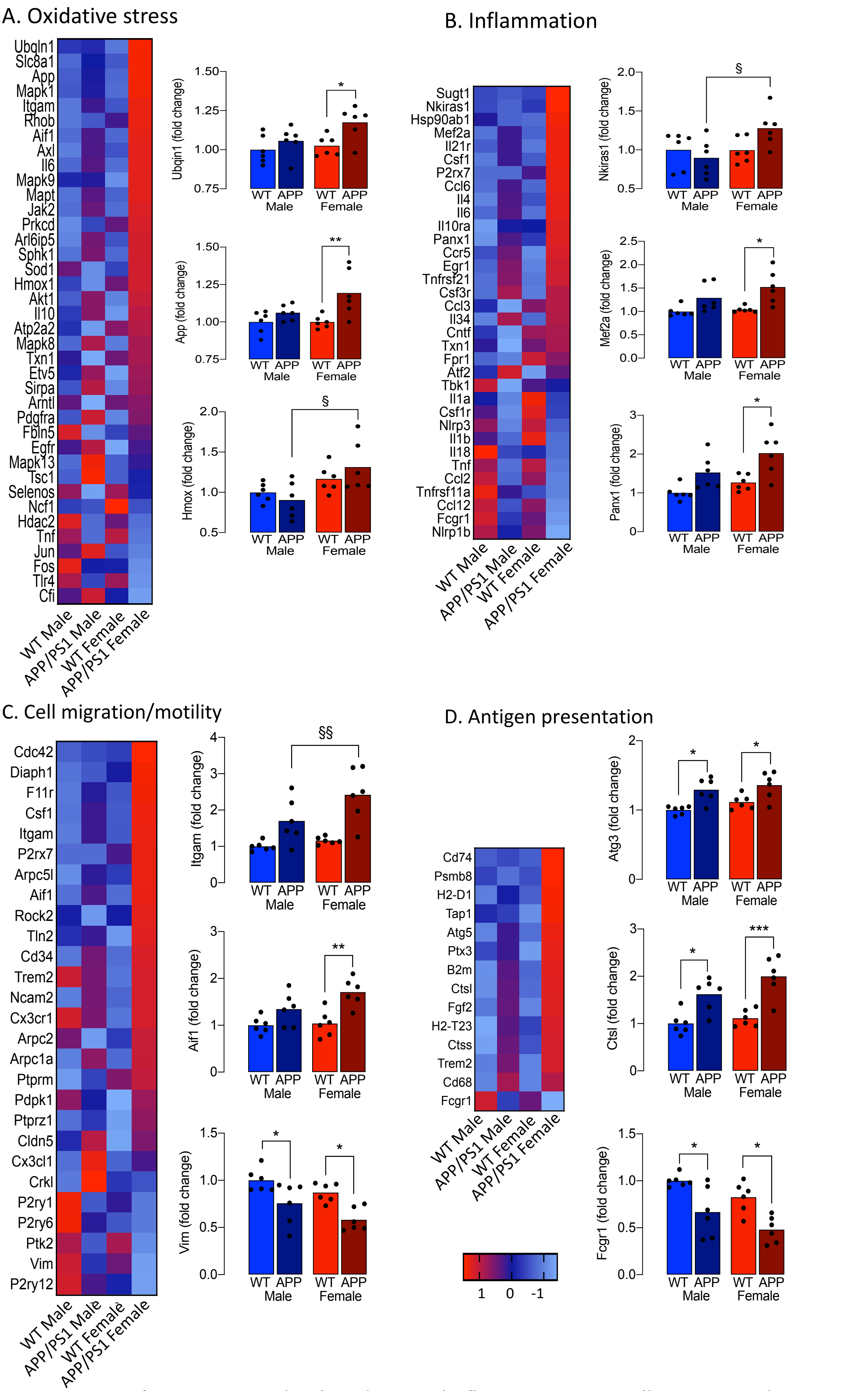

Supplementary Figure 2. Expression of genes associated with oxidative and inflammatory stress, cell migration and antigen presentation is sex-related in microglia from APP/PS1 mice.

A significant main effect of genotype was identified in *Itgam, Atg3, Mef2a, Aif1, Ctsl, Panx1, Vim, Fcgr1* ($p < 0.001$), *App* ($p < 0.01$) and *Ubiqin1* ($p < 0.05$) and a significant main effect of sex was observed in *Hmox* ($p < 0.01$), *Itgam, Panx1, Vim and Fcgr1* ($p < 0.05$). Significant genotype-related increases were observed in several genes in APP/PS1 male, compared with WT male, mice and in APP/PS1 female, compared with WT female, mice as indicated by the asterisks (*$p < 0.05$; **$p < 0.01$; ***$p < 0.001$; n=5 or 6).

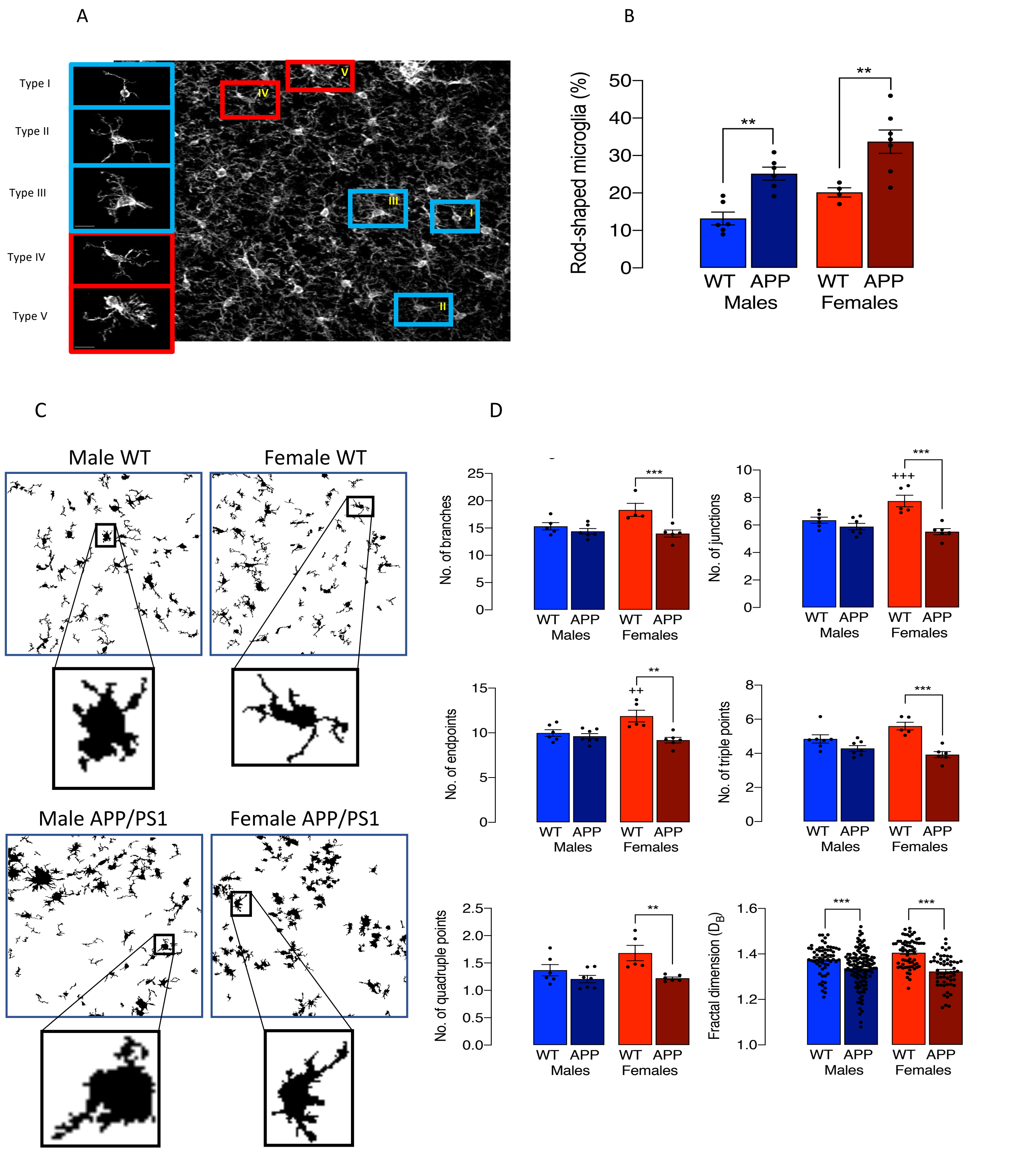

Supplementary Figure 3 : Evidence of genotype- and sex-related changes in microglial morphology.

A. A section through brain of APP/PS1 mouse identifies microglia Type I-V; rod-shaped Types IV and V are shown.

B. The proportion of rod-shaped microglia is significantly increased in cortex of APP/PS1, compared with WT, mice (***$p < 0.001$). Significant main effects of genotype ($p < 0.001$) and sex ($p < 0.01$) were revealed by 2-way ANOVA.

C,D. Masks were prepared for analysis of microglial complexity. The mean number of branches, junctions, endpoints and triple and quadruple points were assessed from masks. A genotype-related decrease in all measures was identified in female APP/PS1, compared with female WT mice and a decrease in fractal dimension was also observed in male APP/PS1, compared with male WT mice (**$p < 0.01$; ***$p < 0.001$).

Data, expressed as means ± SEM (n=5 or 6), were analysed by 2-way ANOVA and Tukey's posthoc multiple comparison test.

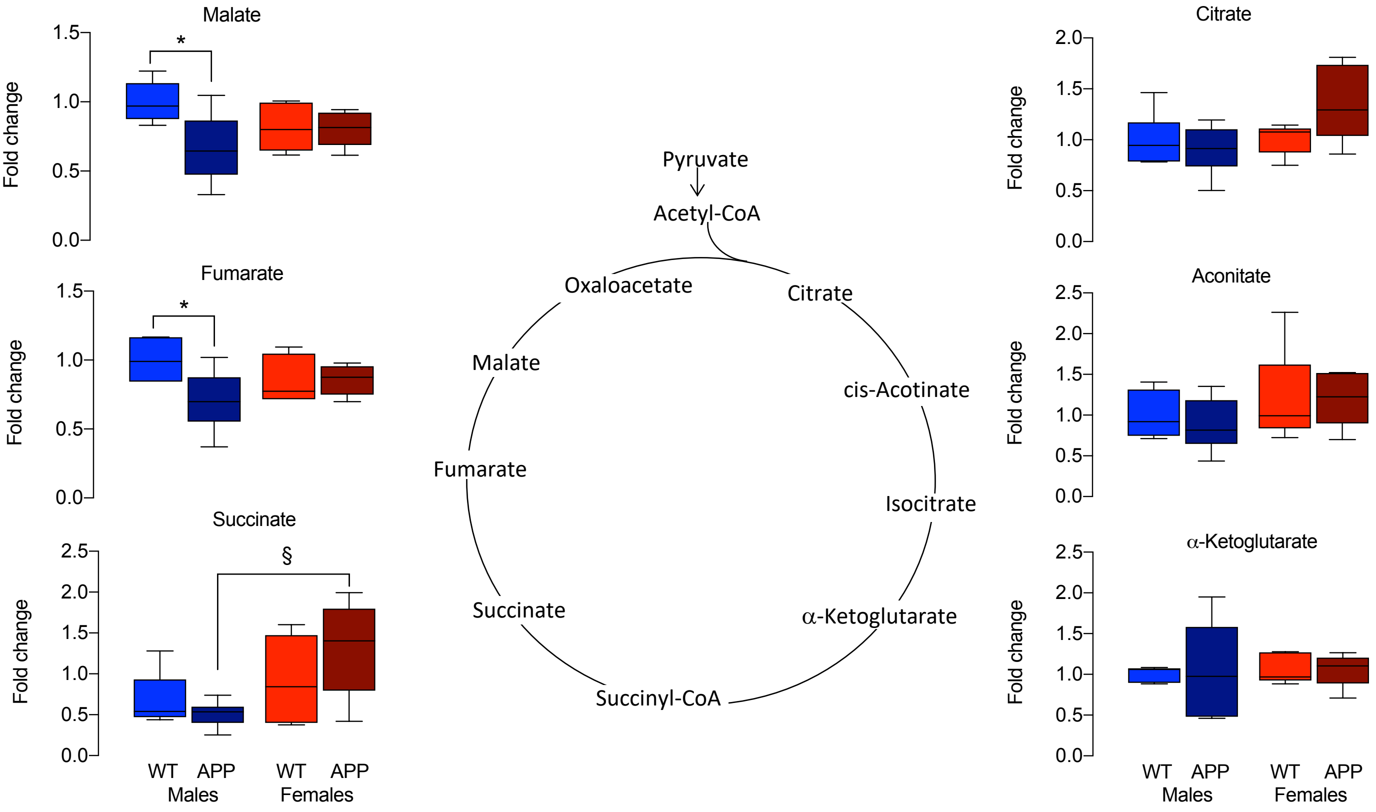

Supplementary Figure 4: Metabolomic analysis of TCA intermediates

Metabolomic analysis revealed that fumarate and malate were decreased in microglia from male APP/PS1, compared with WT, mice (*$p < 0.05$). Succinate was increased in microglia from female, compared with male, APP/PS1 mice ($^{§}p < 0.05$). No significant changes in citrate, cis-aconitate or a-ketoglutarate were observed. Data, expressed as means ± SEM (n=5 or 6), were analysed by 2-way ANOVA and Tukey's posthoc multiple comparison test.

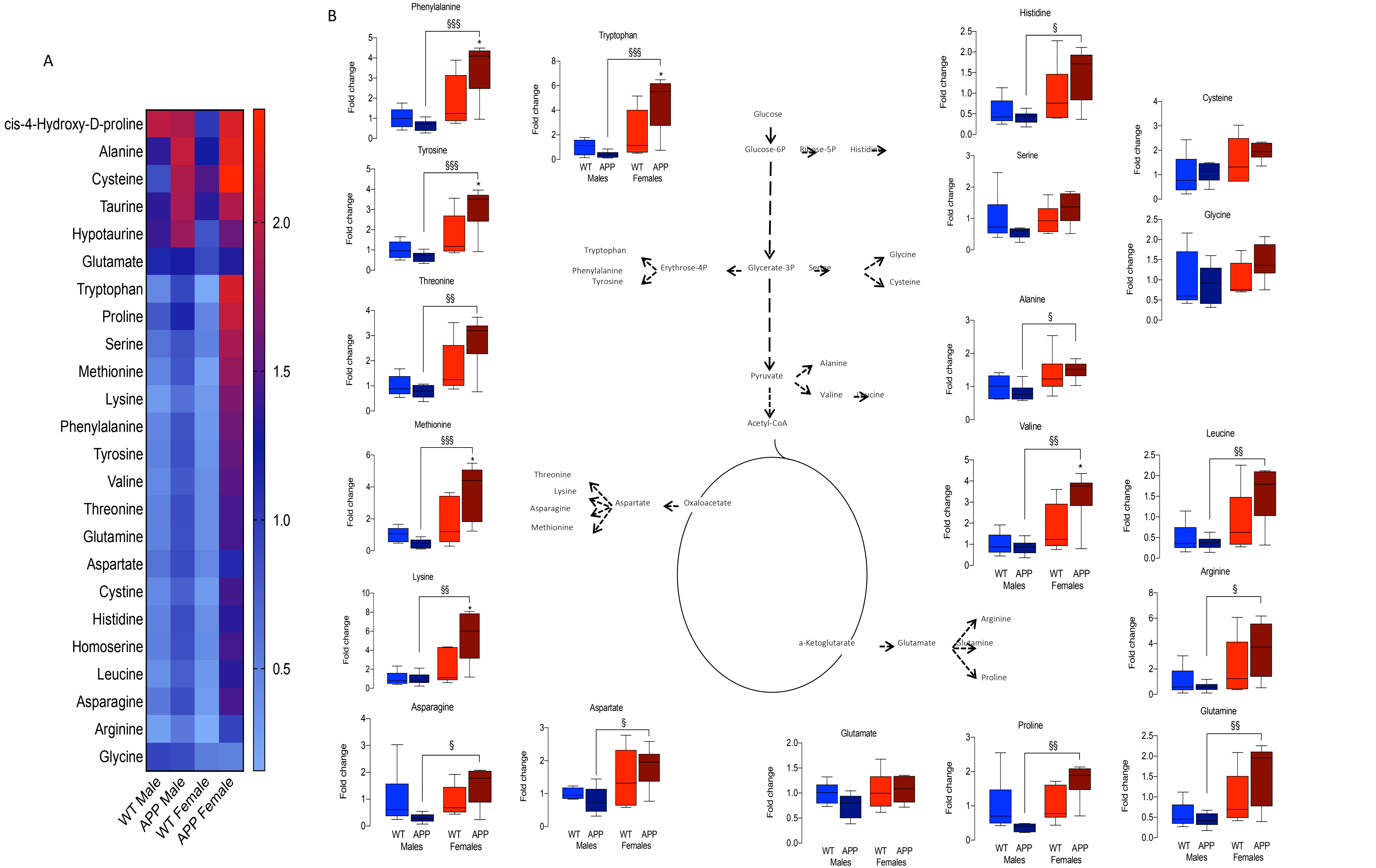

Supplementary Figure 5: Metabolomic analysis of TCA intermediates

Metabolomic analysis revealed that fumarate and malate were decreased in microglia from male APP/PS1, compared with WT, mice (*$p < 0.05$). Succinate was increased in microglia from female, compared with male, APP/PS1 mice ($^{§}p < 0.05$). No significant changes in citrate, cis-aconitate or a-ketoglutarate were observed. Data, expressed as means ± SEM (n=5 or 6), were analysed by 2-way ANOVA and Tukey's posthoc multiple comparison test.

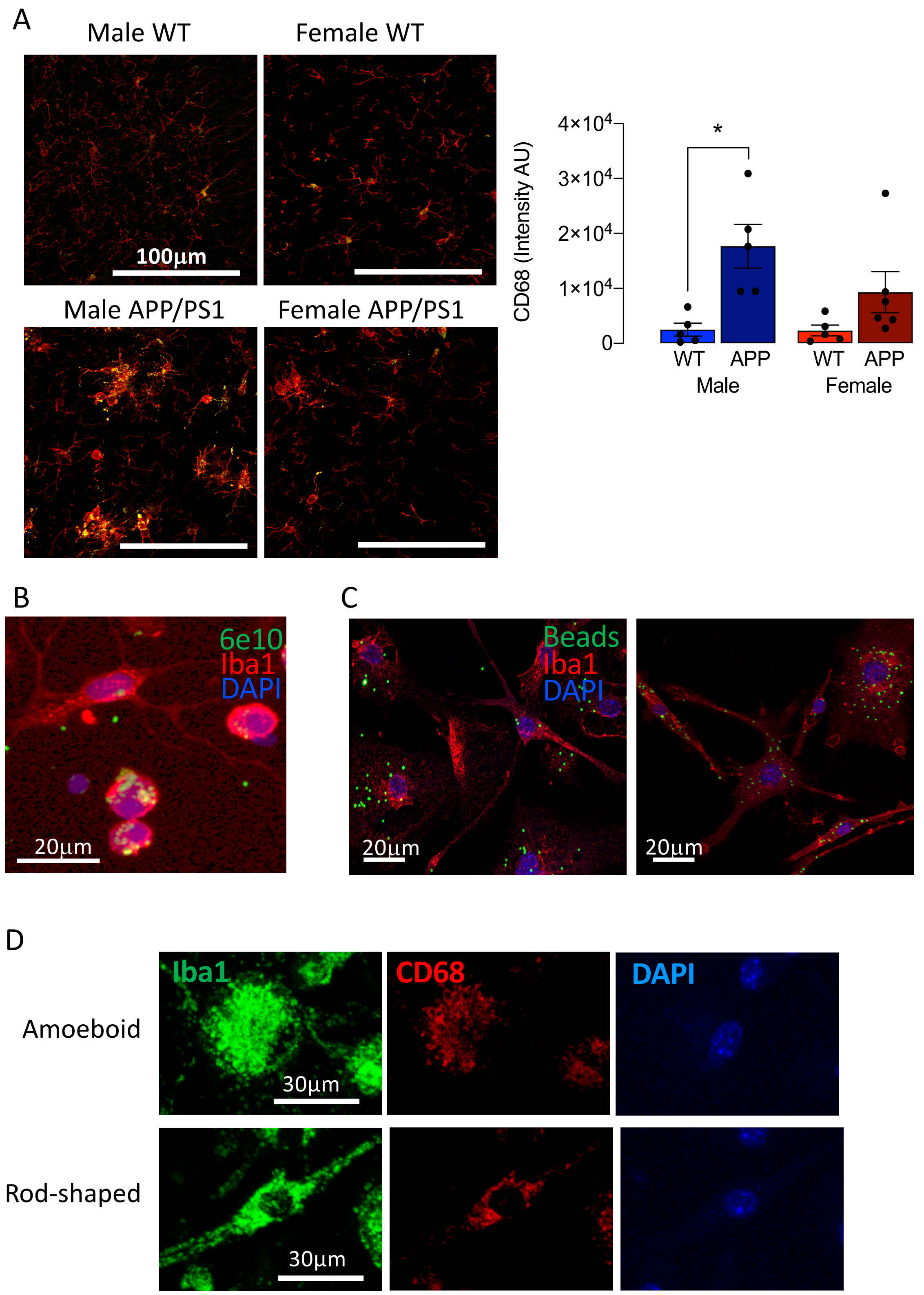

Supplementary Figure 6. Evidence of sex-related changes in CD68 that may reflect altered phagocytic function.
A. CD68 immunoreactivity in hippocampus of male APP/PS1 mice was more marked than in male WT mice and female APP/PS1 mice. Analysis of the mean data revealed a significant main effect of genotype ($p < 0.01$; n=5-6) on immunoreactivity and *post hoc* analysis identified significant genotype-related differences in male mice (*$p < 0.01$; ***$p < 0.001$). B,C. Uptake of Aβ, assessed by 6e10 staining (B) and fluorescently labelled beads (C) was more marked in amoeboid microglia compared with microglia with processes. D. CD68 staining was more pronounced in amoeboid microglia compared with rod-shaped microglia. For A sections were prepared from 18 month-old mice, for B and C staining was assessed in cultured microglia from neonates and isolated microglia from aged mice respectively, and for D staining was assessed in isolated microglia prepared from adult mice.
