# Supplementary material for: Microglial metabolism is a pivotal factor in sexual dimorphism in Alzheimer’s disease
Source: Commun Biol. 2021 Jun 10;4:711. doi: 10.1038/s42003-021-02259-y (PMC8192523; doi:10.1038/s42003-021-02259-y)
Supplement: Supplementary file 3 — Reporting Summary [file 42003_2021_2259_MOESM3_ESM.pdf]

nature research

Corresponding author(s): Marina Lynch

Last updated by author(s): 2021/5/14

Reporting Summary

Nature Research wishes to improve the reproducibility of the work that we publish. This form provides structure for consistency and transparency in reporting. For further information on Nature Research policies, see our Editorial Policies and the Editorial Policy Checklist.

Statistics

For all statistical analyses, confirm that the following items are present in the figure legend, table legend, main text, or Methods section.

n/a	Confirmed	
☐	☒	The exact sample size (n) for each experimental group/condition, given as a discrete number and unit of measurement
☐	☒	A statement on whether measurements were taken from distinct samples or whether the same sample was measured repeatedly
☐	☒	The statistical test(s) used AND whether they are one- or two-sided<br>*Only common tests should be described solely by name; describe more complex techniques in the Methods section.*
☐	☒	A description of all covariates tested
☒	☐	A description of any assumptions or corrections, such as tests of normality and adjustment for multiple comparisons
☐	☒	A full description of the statistical parameters including central tendency (e.g. means) or other basic estimates (e.g. regression coefficient) AND variation (e.g. standard deviation) or associated estimates of uncertainty (e.g. confidence intervals)
☒	☐	For null hypothesis testing, the test statistic (e.g. F, t, r) with confidence intervals, effect sizes, degrees of freedom and P value noted<br>*Give P values as exact values whenever suitable.*
☒	☐	For Bayesian analysis, information on the choice of priors and Markov chain Monte Carlo settings
☒	☐	For hierarchical and complex designs, identification of the appropriate level for tests and full reporting of outcomes
☒	☐	Estimates of effect sizes (e.g. Cohen's d, Pearson's r), indicating how they were calculated

Our web collection on statistics for biologists contains articles on many of the points above.

Software and code

Policy information about availability of computer code

Data collection	Not applicable
Data analysis	The Nanostring data were assessed using nSolver software and all other data were assessed using Prism 9.

For manuscripts utilizing custom algorithms or software that are central to the research but not yet described in published literature, software must be made available to editors and reviewers. We strongly encourage code deposition in a community repository (e.g. GitHub). See the Nature Research guidelines for submitting code & software for further information.

Data

Policy information about availability of data

All manuscripts must include a data availability statement. This statement should provide the following information, where applicable:

- Accession codes, unique identifiers, or web links for publicly available datasets
- A list of figures that have associated raw data
- A description of any restrictions on data availability

All data are available on request and, when published, the Nanostring data will be deposited in an appropriate repository

Field-specific reporting

Please select the one below that is the best fit for your research. If you are not sure, read the appropriate sections before making your selection.

☒ Life sciences ☐ Behavioural & social sciences ☐ Ecological, evolutionary & environmental sciences

For a reference copy of the document with all sections, see nature.com/documents/nr-reporting-summary-flat.pdf

Life sciences study design

All studies must disclose on these points even when the disclosure is negative.

Sample size	Sample sizes were chosen based on findings from similar previous experiments.
Data exclusions	Any data exclusions, which are identified in the EXCEL sheet were based on outlier analysis in the Prism 9 package
Replication	Data were obtained from (a) several preparations of microglia isolated from mice or (b) sections prepared from several mice. In some cases, reproducibility was assessed by more than one individual undertaking the experiments. We have no reason to believe that reproducibility is an issue.
Randomization	Animals were allocated to experimental groups based on genotype and sex so randomization is limited in this study. Litter mates were used as controls (ie WT mice). Post-mortem tissue from AD patients and age-matched controls were allocated to separate groups.
Blinding	Blinding is constrained for those culling mice because mice were caged by genotype and sex. Experimenters were unaware of the origin of cells and sample during analysis although no formal arrangements were in place.

Reporting for specific materials, systems and methods

We require information from authors about some types of materials, experimental systems and methods used in many studies. Here, indicate whether each material, system or method listed is relevant to your study. If you are not sure if a list item applies to your research, read the appropriate section before selecting a response.

Materials & experimental systems

n/a	Involved in the study	
☐	☒	Antibodies
☒	☐	Eukaryotic cell lines
☒	☐	Palaeontology and archaeology
☐	☒	Animals and other organisms
☐	☒	Human research participants
☒	☐	Clinical data
☒	☐	Dual use research of concern

Methods

n/a	Involved in the study	
☒	☐	ChIP-seq
☒	☐	Flow cytometry
☒	☐	MRI-based neuroimaging

Antibodies

Antibodies used	The antibodies used and details are in the attached table
Validation	Antibodies were chosen in the first instance based on manufacturer's website but most have been used in different studies in the lab for several years. All were checked for specificity in microglia.

Animals and other organisms

Policy information about studies involving animals; ARRIVE guidelines recommended for reporting animal research

Laboratory animals	Male and female transgenic mice that overexpress mutant amyloid precursor protein (APPswe) and presenilin 1 (PSEN1ΔE9; APP/PS1 mice, B6;C3-Tg (APPswePSEN1ΔE9)85Dbo/Mmjax MMRRC (RRID:MMRRC_034829-JAX) and littermate controls were used in these studies. The mice were bred and maintained in the facility in Trinity College Dublin
Wild animals	*Provide details on animals observed in or captured in the field; report species, sex and age where possible. Describe how animals were caught and transported and what happened to captive animals after the study (if killed, explain why and describe method; if released, say where and when) OR state that the study did not involve wild animals.*
Field-collected samples	*For laboratory work with field-collected samples, describe all relevant parameters such as housing, maintenance, temperature, photoperiod and end-of-experiment protocol OR state that the study did not involve samples collected from the field.*

Ethics oversight	*Identify the organization(s) that approved or provided guidance on the study protocol, OR state that no ethical approval or guidance was required and explain why not.*

Note that full information on the approval of the study protocol must also be provided in the manuscript.

Human research participants

Policy information about studies involving human research participants

Population characteristics	Table 1 in the manuscript describes the samples. Post-mortem samples of hippocampus and parietal cortex were obtained from 5 male (aged 71-81 years) and 4 female (75-82 years) AD patients and 5 male (75-82 years) and 4 female (75-80 years) age-matched controls.
Recruitment	Individuals living in the Netherlands register as a brain donor. By signing the Informed Consent form, donors gives permission for post-mortem brain autopsy and use of their brain material and medical records for research purposes. The informed consent form of the Netherlands Brain Bank meets all current legal and ethical requirements for brain autopsy, tissue storage and use of tissue and clinical data for scientific research worldwide.
Ethics oversight	The Netherlands Brain Bank provided the samples, which were collected from volunteers with appropriate ethical approval.

Note that full information on the approval of the study protocol must also be provided in the manuscript.
